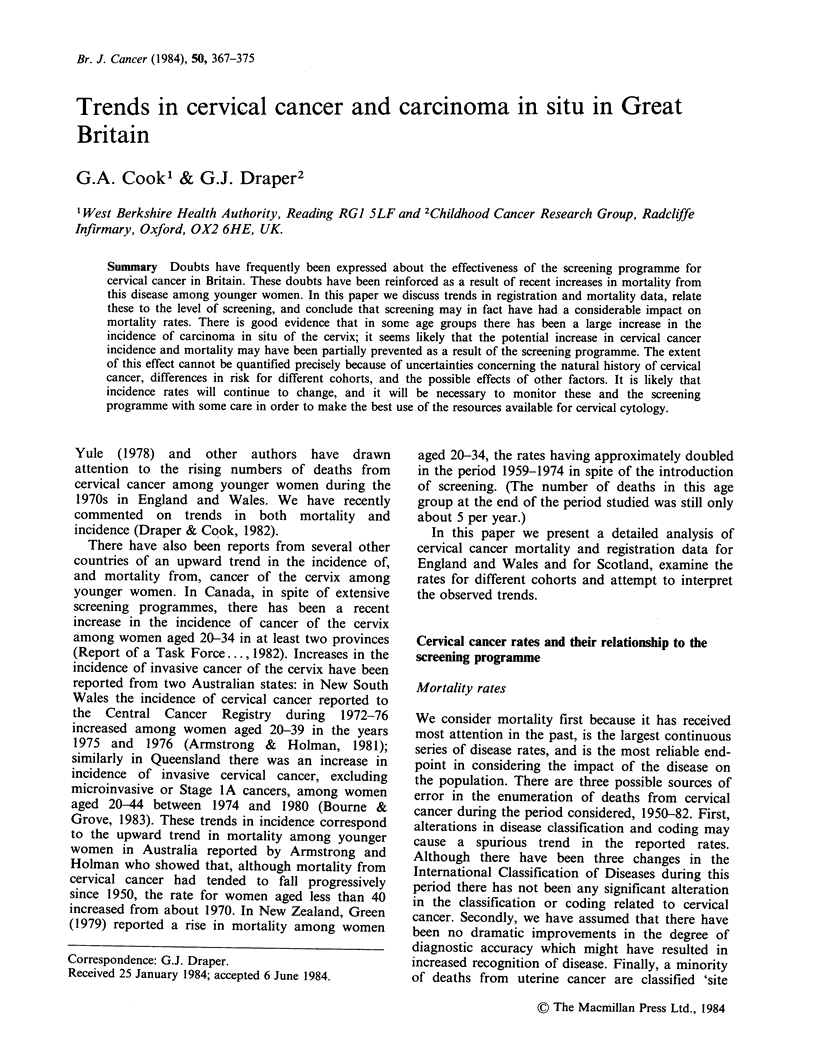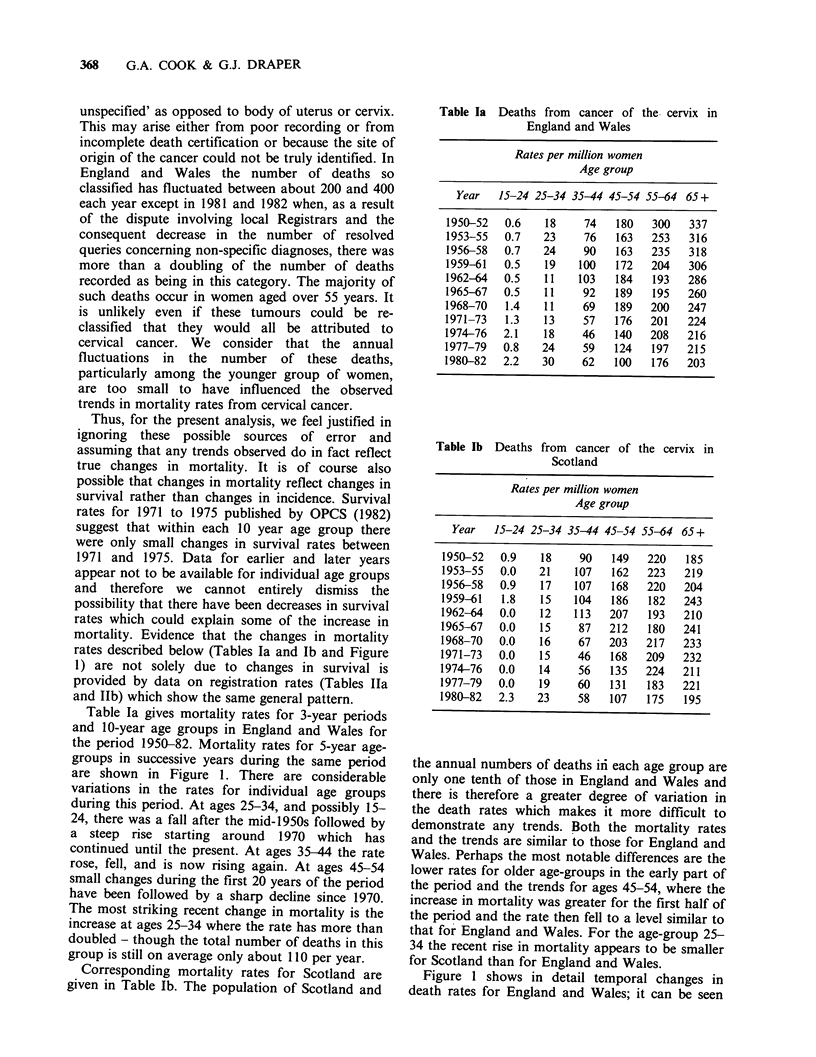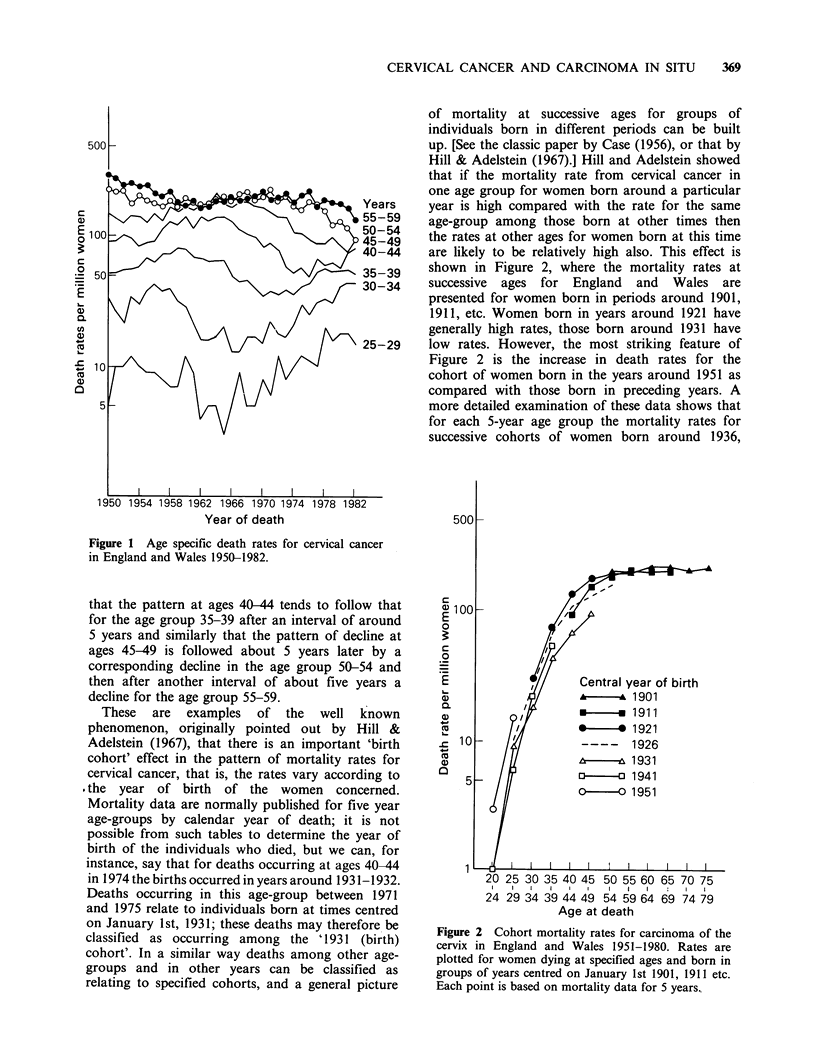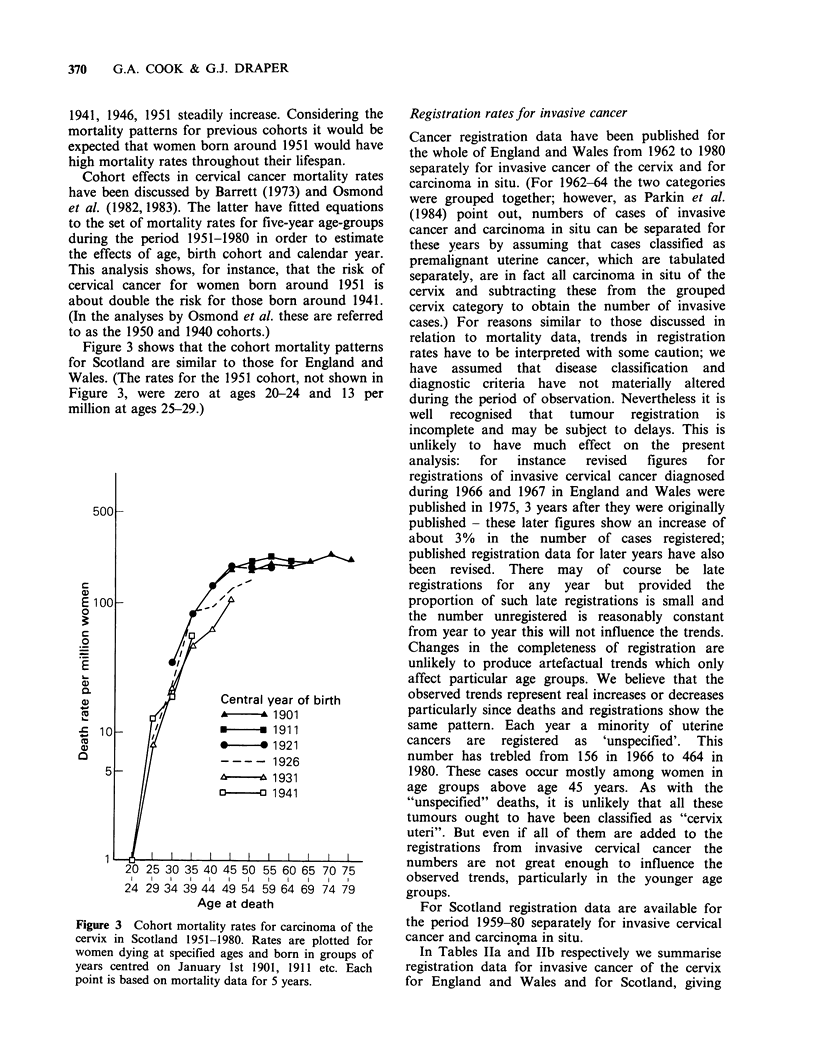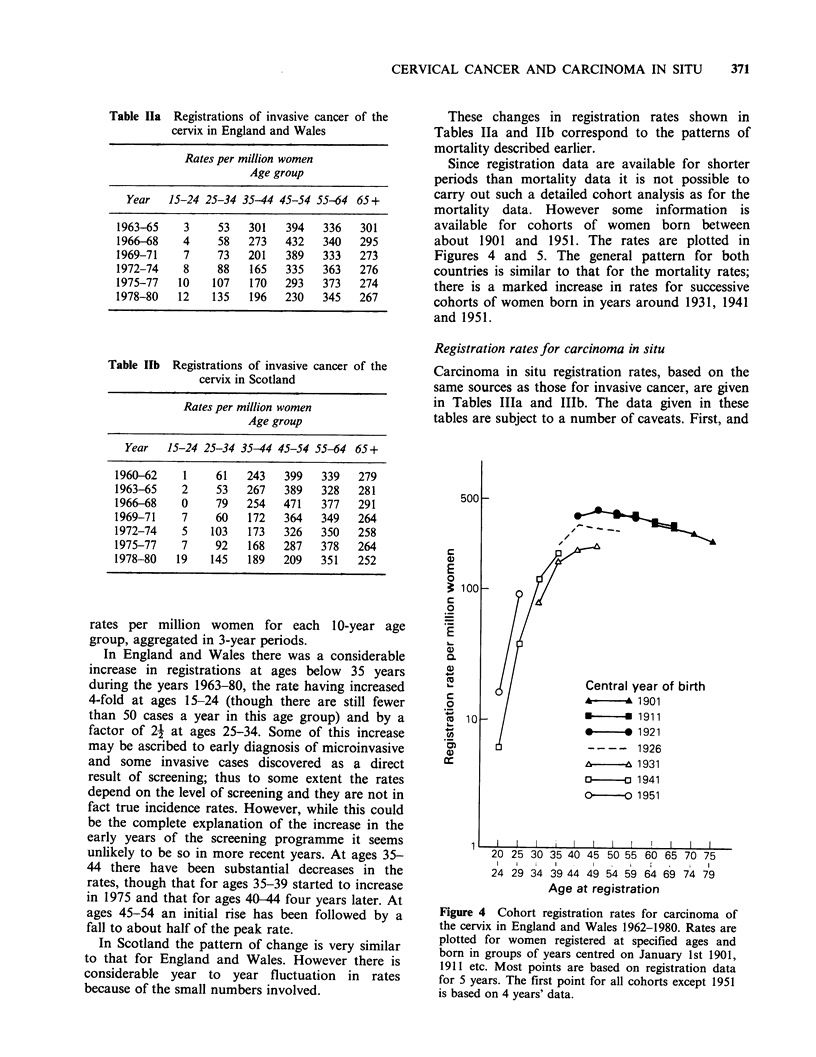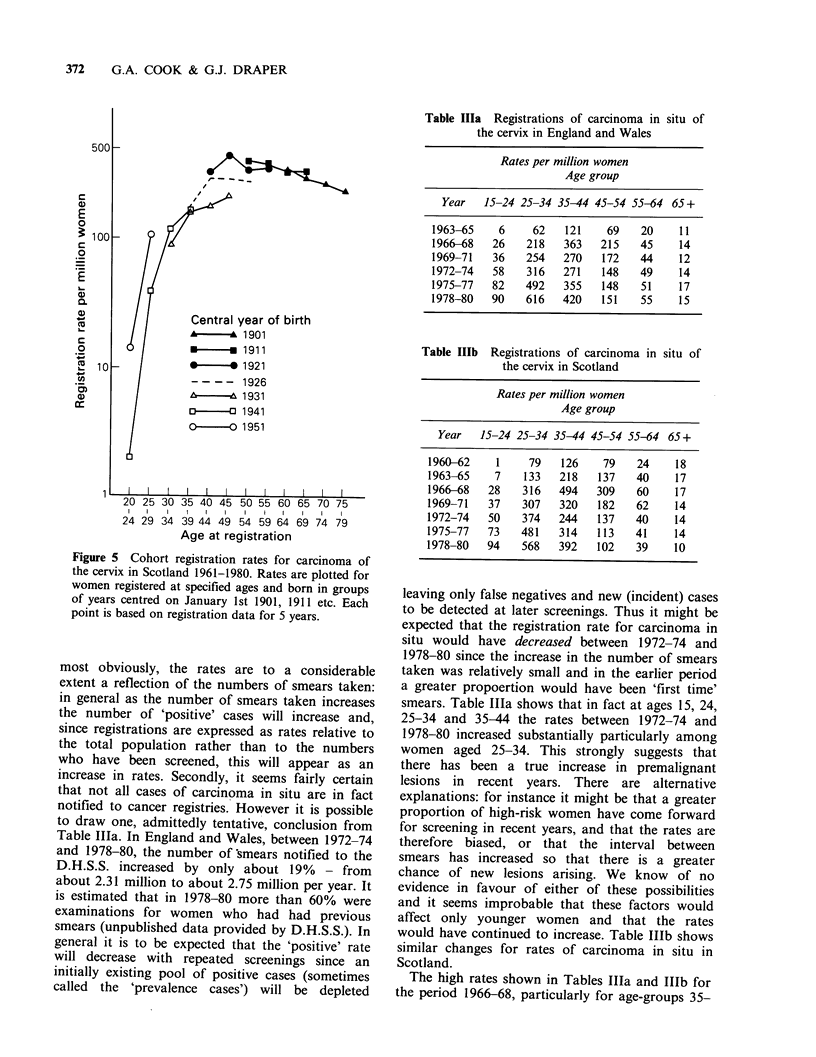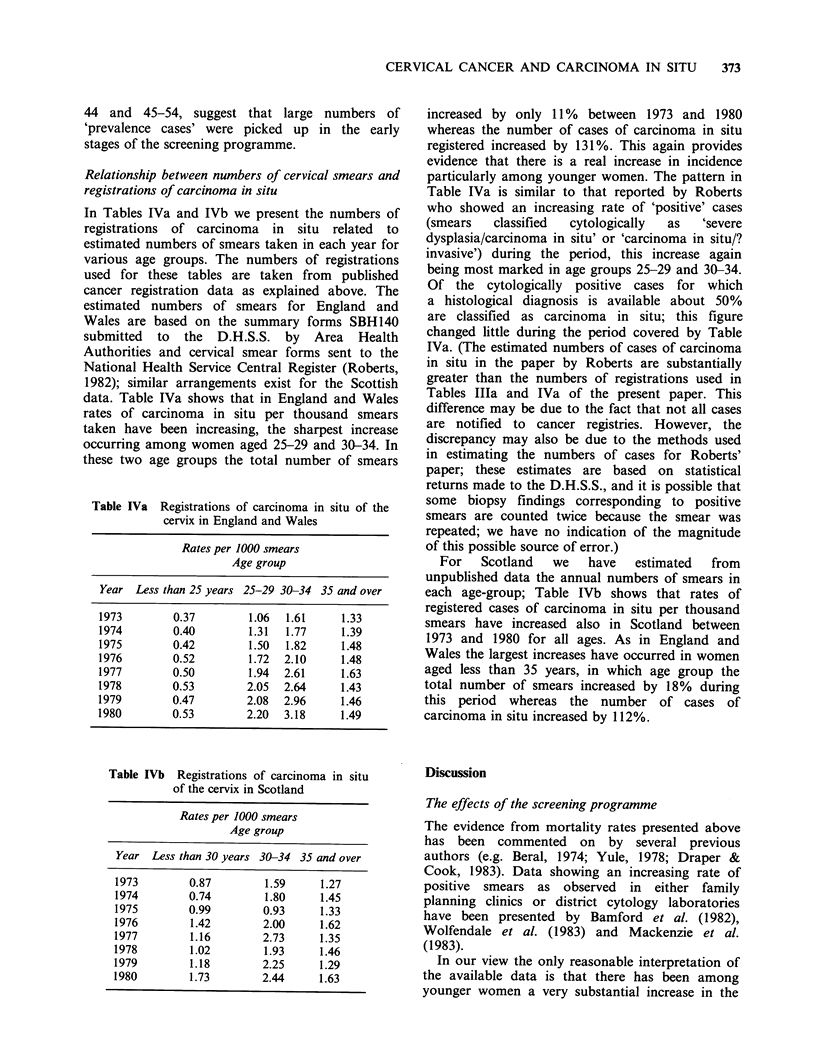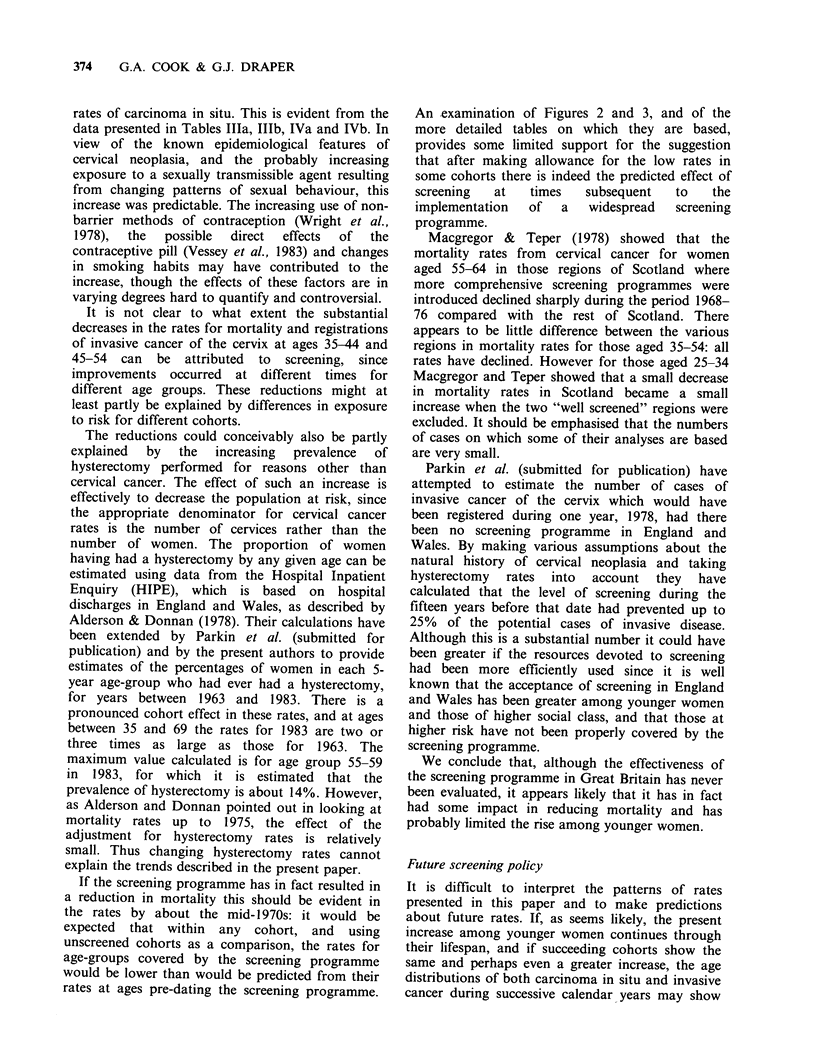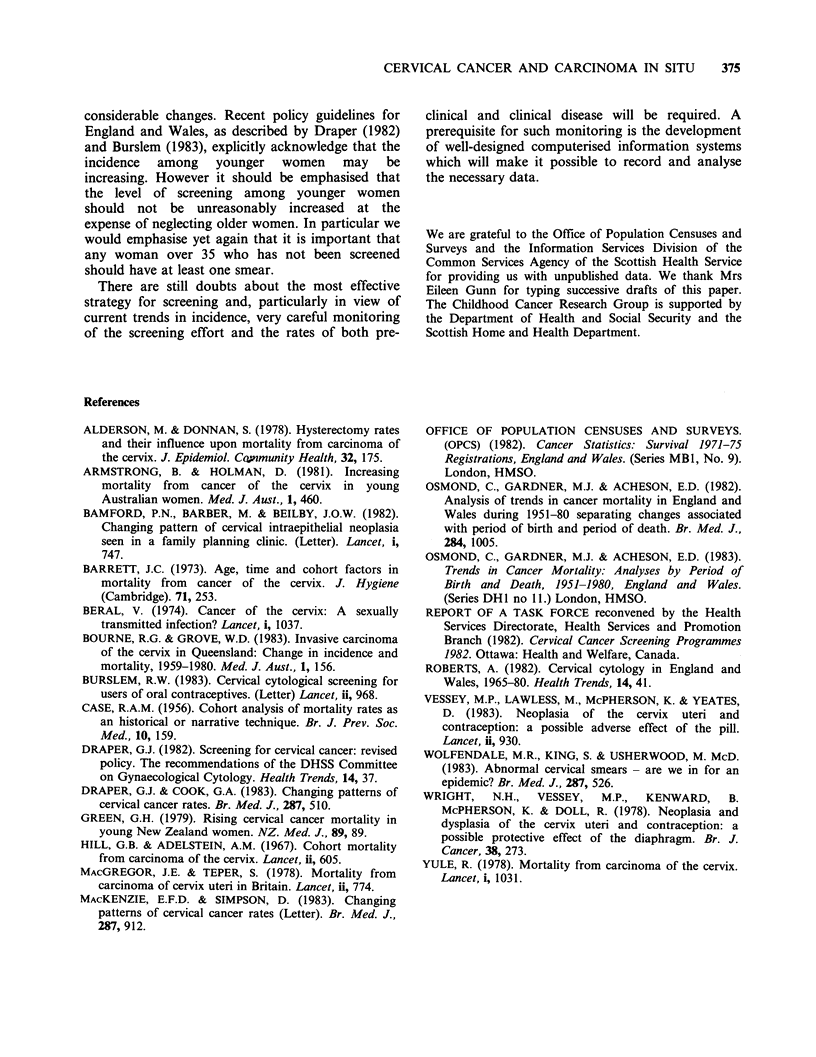# Trends in cervical cancer and carcinoma in situ in Great Britain.

**DOI:** 10.1038/bjc.1984.185

**Published:** 1984-09

**Authors:** G. A. Cook, G. J. Draper

## Abstract

Doubts have frequently been expressed about the effectiveness of the screening programme for cervical cancer in Britain. These doubts have been reinforced as a result of recent increases in mortality from this disease among younger women. In this paper we discuss trends in registration and mortality data, relate these to the level of screening, and conclude that screening may in fact have had a considerable impact on mortality rates. There is good evidence that in some age groups there has been a large increase in the incidence of carcinoma in situ of the cervix; it seems likely that the potential increase in cervical cancer incidence and mortality may have been partially prevented as a result of the screening programme. The extent of this effect cannot be quantified precisely because of uncertainties concerning the natural history of cervical cancer, differences in risk for different cohorts, and the possible effects of other factors. It is likely that incidence rates will continue to change, and it will be necessary to monitor these and the screening programme with some care in order to make the best use of the resources available for cervical cytology.


Br. J. Cancer (1984), 50, 367–375

### Trends in cervical cancer and carcinoma in situ in Great Britain

G.A. Cook[1] & G.J. Draper[2]

[1]*West Berkshire Health Authority, Reading RG1 5LF and* [2]*Childhood Cancer Research Group, Radcliffe Infirmary, Oxford, OX2 6HE, UK.*

**Summary** Doubts have frequently been expressed about the effectiveness of the screening programme for cervical cancer in Britain. These doubts have been reinforced as a result of recent increases in mortality from this disease among younger women. In this paper we discuss trends in registration and mortality data, relate these to the level of screening, and conclude that screening may in fact have had a considerable impact on mortality rates. There is good evidence that in some age groups there has been a large increase in the incidence of carcinoma in situ of the cervix; it seems likely that the potential increase in cervical cancer incidence and mortality may have been partially prevented as a result of the screening programme. The extent of this effect cannot be quantified precisely because of uncertainties concerning the natural history of cervical cancer, differences in risk for different cohorts, and the possible effects of other factors. It is likely that incidence rates will continue to change, and it will be necessary to monitor these and the screening programme with some care in order to make the best use of the resources available for cervical cytology.

Yule (1978) and other authors have drawn attention to the rising numbers of deaths from cervical cancer among younger women during the 1970s in England and Wales. We have recently commented on trends in both mortality and incidence (Draper & Cook, 1982).

There have also been reports from several other countries of an upward trend in the incidence of, and mortality from, cancer of the cervix among younger women. In Canada, in spite of extensive screening programmes, there has been a recent increase in the incidence of cancer of the cervix among women aged 20–34 in at least two provinces (Report of a Task Force..., 1982). Increases in the incidence of invasive cancer of the cervix have been reported from two Australian states: in New South Wales the incidence of cervical cancer reported to the Central Cancer Registry during 1972–76 increased among women aged 20–39 in the years 1975 and 1976 (Armstrong & Holman, 1981); similarly in Queensland there was an increase in incidence of invasive cervical cancer, excluding microinvasive or Stage 1A cancers, among women aged 20–44 between 1974 and 1980 (Bourne & Grove, 1983). These trends in incidence correspond to the upward trend in mortality among younger women in Australia reported by Armstrong and Holman who showed that, although mortality from cervical cancer had tended to fall progressively since 1950, the rate for women aged less than 40 increased from about 1970. In New Zealand, Green (1979) reported a rise in mortality among women aged 20–34, the rates having approximately doubled in the period 1959–1974 in spite of the introduction of screening. (The number of deaths in this age group at the end of the period studied was still only about 5 per year.)

In this paper we present a detailed analysis of cervical cancer mortality and registration data for England and Wales and for Scotland, examine the rates for different cohorts and attempt to interpret the observed trends.

### Cervical cancer rates and their relationship to the screening programme

### *Mortality rates*

We consider mortality first because it has received most attention in the past, is the largest continuous series of disease rates, and is the most reliable end-point in considering the impact of the disease on the population. There are three possible sources of error in the enumeration of deaths from cervical cancer during the period considered, 1950–82. First, alterations in disease classification and coding may cause a spurious trend in the reported rates. Although there have been three changes in the International Classification of Diseases during this period there has not been any significant alteration in the classification or coding related to cervical cancer. Secondly, we have assumed that there have been no dramatic improvements in the degree of diagnostic accuracy which might have resulted in increased recognition of disease. Finally, a minority of deaths from uterine cancer are classified 'site

Correspondence: G.J. Draper.
Received 25 January 1984; accepted 6 June 1984.

© The Macmillan Press Ltd., 1984

unspecified' as opposed to body of uterus or cervix. This may arise either from poor recording or from incomplete death certification or because the site of origin of the cancer could not be truly identified. In England and Wales the number of deaths so classified has fluctuated between about 200 and 400 each year except in 1981 and 1982 when, as a result of the dispute involving local Registrars and the consequent decrease in the number of resolved queries concerning non-specific diagnoses, there was more than a doubling of the number of deaths recorded as being in this category. The majority of such deaths occur in women aged over 55 years. It is unlikely even if these tumours could be re-classified that they would all be attributed to cervical cancer. We consider that the annual fluctuations in the number of these deaths, particularly among the younger group of women, are too small to have influenced the observed trends in mortality rates from cervical cancer.

Thus, for the present analysis, we feel justified in ignoring these possible sources of error and assuming that any trends observed do in fact reflect true changes in mortality. It is of course also possible that changes in mortality reflect changes in survival rather than changes in incidence. Survival rates for 1971 to 1975 published by OPCS (1982) suggest that within each 10 year age group there were only small changes in survival rates between 1971 and 1975. Data for earlier and later years appear not to be available for individual age groups and therefore we cannot entirely dismiss the possibility that there have been decreases in survival rates which could explain some of the increase in mortality. Evidence that the changes in mortality rates described below (Tables Ia and Ib and Figure 1) are not solely due to changes in survival is provided by data on registration rates (Tables IIa and IIb) which show the same general pattern.

Table Ia gives mortality rates for 3-year periods and 10-year age groups in England and Wales for the period 1950–82. Mortality rates for 5-year age-groups in successive years during the same period are shown in Figure 1. There are considerable variations in the rates for individual age groups during this period. At ages 25–34, and possibly 15–24, there was a fall after the mid-1950s followed by a steep rise starting around 1970 which has continued until the present. At ages 35–44 the rate rose, fell, and is now rising again. At ages 45–54 small changes during the first 20 years of the period have been followed by a sharp decline since 1970. The most striking recent change in mortality is the increase at ages 25–34 where the rate has more than doubled – though the total number of deaths in this group is still on average only about 110 per year.

Corresponding mortality rates for Scotland are given in Table Ib. The population of Scotland and the annual numbers of deaths in each age group are only one tenth of those in England and Wales and there is therefore a greater degree of variation in the death rates which makes it more difficult to demonstrate any trends. Both the mortality rates and the trends are similar to those for England and Wales. Perhaps the most notable differences are the lower rates for older age-groups in the early part of the period and the trends for ages 45–54, where the increase in mortality was greater for the first half of the period and the rate then fell to a level similar to that for England and Wales. For the age-group 25–34 the recent rise in mortality appears to be smaller for Scotland than for England and Wales.

Figure 1 shows in detail temporal changes in death rates for England and Wales; it can be seen

**Table Ia** Deaths from cancer of the cervix in England and Wales

| | *Rates per million women* | | | | | |
|---|---|---|---|---|---|---|
| | *Age group* | | | | | |
| *Year* | *15–24* | *25–34* | *35–44* | *45–54* | *55–64* | *65+* |
| 1950–52 | 0.6 | 18 | 74 | 180 | 300 | 337 |
| 1953–55 | 0.7 | 23 | 76 | 163 | 253 | 316 |
| 1956–58 | 0.7 | 24 | 90 | 163 | 235 | 318 |
| 1959–61 | 0.5 | 19 | 100 | 172 | 204 | 306 |
| 1962–64 | 0.5 | 11 | 103 | 184 | 193 | 286 |
| 1965–67 | 0.5 | 11 | 92 | 189 | 195 | 260 |
| 1968–70 | 1.4 | 11 | 69 | 189 | 200 | 247 |
| 1971–73 | 1.3 | 13 | 57 | 176 | 201 | 224 |
| 1974–76 | 2.1 | 18 | 46 | 140 | 208 | 216 |
| 1977–79 | 0.8 | 24 | 59 | 124 | 197 | 215 |
| 1980–82 | 2.2 | 30 | 62 | 100 | 176 | 203 |

**Table Ib** Deaths from cancer of the cervix in Scotland

| | *Rates per million women* | | | | | |
|---|---|---|---|---|---|---|
| | *Age group* | | | | | |
| *Year* | *15–24* | *25–34* | *35–44* | *45–54* | *55–64* | *65+* |
| 1950–52 | 0.9 | 18 | 90 | 149 | 220 | 185 |
| 1953–55 | 0.0 | 21 | 107 | 162 | 223 | 219 |
| 1956–58 | 0.9 | 17 | 107 | 168 | 220 | 204 |
| 1959–61 | 1.8 | 15 | 104 | 186 | 182 | 243 |
| 1962–64 | 0.0 | 12 | 113 | 207 | 193 | 210 |
| 1965–67 | 0.0 | 15 | 87 | 212 | 180 | 241 |
| 1968–70 | 0.0 | 16 | 67 | 203 | 217 | 233 |
| 1971–73 | 0.0 | 15 | 46 | 168 | 209 | 232 |
| 1974–76 | 0.0 | 14 | 56 | 135 | 224 | 211 |
| 1977–79 | 0.0 | 19 | 60 | 131 | 183 | 221 |
| 1980–82 | 2.3 | 23 | 58 | 107 | 175 | 195 |

**Figure 1** Age specific death rates for cervical cancer in England and Wales 1950–1982.

of mortality at successive ages for groups of individuals born in different periods can be built up. [See the classic paper by Case (1956), or that by Hill & Adelstein (1967).] Hill and Adelstein showed that if the mortality rate from cervical cancer in one age group for women born around a particular year is high compared with the rate for the same age-group among those born at other times then the rates at other ages for women born at this time are likely to be relatively high also. This effect is shown in Figure 2, where the mortality rates at successive ages for England and Wales are presented for women born in periods around 1901, 1911, etc. Women born in years around 1921 have generally high rates, those born around 1931 have low rates. However, the most striking feature of Figure 2 is the increase in death rates for the cohort of women born in the years around 1951 as compared with those born in preceding years. A more detailed examination of these data shows that for each 5-year age group the mortality rates for successive cohorts of women born around 1936, that the pattern at ages 40–44 tends to follow that for the age group 35–39 after an interval of around 5 years and similarly that the pattern of decline at ages 45–49 is followed about 5 years later by a corresponding decline in the age group 50–54 and then after another interval of about five years a decline for the age group 55–59.

These are examples of the well known phenomenon, originally pointed out by Hill & Adelstein (1967), that there is an important 'birth cohort' effect in the pattern of mortality rates for cervical cancer, that is, the rates vary according to the year of birth of the women concerned. Mortality data are normally published for five year age-groups by calendar year of death; it is not possible from such tables to determine the year of birth of the individuals who died, but we can, for instance, say that for deaths occurring at ages 40–44 in 1974 the births occurred in years around 1931–1932. Deaths occurring in this age-group between 1971 and 1975 relate to individuals born at times centred on January 1st, 1931; these deaths may therefore be classified as occurring among the '1931 (birth) cohort'. In a similar way deaths among other age-groups and in other years can be classified as relating to specified cohorts, and a general picture

**Figure 2** Cohort mortality rates for carcinoma of the cervix in England and Wales 1951–1980. Rates are plotted for women dying at specified ages and born in groups of years centred on January 1st 1901, 1911 etc. Each point is based on mortality data for 5 years.

1941, 1946, 1951 steadily increase. Considering the mortality patterns for previous cohorts it would be expected that women born around 1951 would have high mortality rates throughout their lifespan.

Cohort effects in cervical cancer mortality rates have been discussed by Barrett (1973) and Osmond *et al.* (1982, 1983). The latter have fitted equations to the set of mortality rates for five-year age-groups during the period 1951–1980 in order to estimate the effects of age, birth cohort and calendar year. This analysis shows, for instance, that the risk of cervical cancer for women born around 1951 is about double the risk for those born around 1941. (In the analyses by Osmond *et al.* these are referred to as the 1950 and 1940 cohorts.)

Figure 3 shows that the cohort mortality patterns for Scotland are similar to those for England and Wales. (The rates for the 1951 cohort, not shown in Figure 3, were zero at ages 20–24 and 13 per million at ages 25–29.)

**Figure 3** Cohort mortality rates for carcinoma of the cervix in Scotland 1951–1980. Rates are plotted for women dying at specified ages and born in groups of years centred on January 1st 1901, 1911 etc. Each point is based on mortality data for 5 years.

*Registration rates for invasive cancer*

Cancer registration data have been published for the whole of England and Wales from 1962 to 1980 separately for invasive cancer of the cervix and for carcinoma in situ. (For 1962–64 the two categories were grouped together; however, as Parkin *et al.* (1984) point out, numbers of cases of invasive cancer and carcinoma in situ can be separated for these years by assuming that cases classified as premalignant uterine cancer, which are tabulated separately, are in fact all carcinoma in situ of the cervix and subtracting these from the grouped cervix category to obtain the number of invasive cases.) For reasons similar to those discussed in relation to mortality data, trends in registration rates have to be interpreted with some caution; we have assumed that disease classification and diagnostic criteria have not materially altered during the period of observation. Nevertheless it is well recognised that tumour registration is incomplete and may be subject to delays. This is unlikely to have much effect on the present analysis: for instance revised figures for registrations of invasive cervical cancer diagnosed during 1966 and 1967 in England and Wales were published in 1975, 3 years after they were originally published – these later figures show an increase of about 3% in the number of cases registered; published registration data for later years have also been revised. There may of course be late registrations for any year but provided the proportion of such late registrations is small and the number unregistered is reasonably constant from year to year this will not influence the trends. Changes in the completeness of registration are unlikely to produce artefactual trends which only affect particular age groups. We believe that the observed trends represent real increases or decreases particularly since deaths and registrations show the same pattern. Each year a minority of uterine cancers are registered as ‘unspecified’. This number has trebled from 156 in 1966 to 464 in 1980. These cases occur mostly among women in age groups above age 45 years. As with the “unspecified” deaths, it is unlikely that all these tumours ought to have been classified as “cervix uteri”. But even if all of them are added to the registrations from invasive cervical cancer the numbers are not great enough to influence the observed trends, particularly in the younger age groups.

For Scotland registration data are available for the period 1959–80 separately for invasive cervical cancer and carcinoma in situ.

In Tables IIa and IIb respectively we summarise registration data for invasive cancer of the cervix for England and Wales and for Scotland, giving

**Table IIa** Registrations of invasive cancer of the cervix in England and Wales

| | *Rates per million women* | | | | | |
|---|---|---|---|---|---|---|
| | *Age group* | | | | | |
| *Year* | *15–24* | *25–34* | *35–44* | *45–54* | *55–64* | *65+* |
| 1963–65 | 3 | 53 | 301 | 394 | 336 | 301 |
| 1966–68 | 4 | 58 | 273 | 432 | 340 | 295 |
| 1969–71 | 7 | 73 | 201 | 389 | 333 | 273 |
| 1972–74 | 8 | 88 | 165 | 335 | 363 | 276 |
| 1975–77 | 10 | 107 | 170 | 293 | 373 | 274 |
| 1978–80 | 12 | 135 | 196 | 230 | 345 | 267 |

**Table IIb** Registrations of invasive cancer of the cervix in Scotland

| | *Rates per million women* | | | | | |
|---|---|---|---|---|---|---|
| | *Age group* | | | | | |
| *Year* | *15–24* | *25–34* | *35–44* | *45–54* | *55–64* | *65+* |
| 1960–62 | 1 | 61 | 243 | 399 | 339 | 279 |
| 1963–65 | 2 | 53 | 267 | 389 | 328 | 281 |
| 1966–68 | 0 | 79 | 254 | 471 | 377 | 291 |
| 1969–71 | 7 | 60 | 172 | 364 | 349 | 264 |
| 1972–74 | 5 | 103 | 173 | 326 | 350 | 258 |
| 1975–77 | 7 | 92 | 168 | 287 | 378 | 264 |
| 1978–80 | 19 | 145 | 189 | 209 | 351 | 252 |

rates per million women for each 10-year age group, aggregated in 3-year periods.

In England and Wales there was a considerable increase in registrations at ages below 35 years during the years 1963–80, the rate having increased 4-fold at ages 15–24 (though there are still fewer than 50 cases a year in this age group) and by a factor of $2\frac{1}{2}$ at ages 25–34. Some of this increase may be ascribed to early diagnosis of microinvasive and some invasive cases discovered as a direct result of screening; thus to some extent the rates depend on the level of screening and they are not in fact true incidence rates. However, while this could be the complete explanation of the increase in the early years of the screening programme it seems unlikely to be so in more recent years. At ages 35–44 there have been substantial decreases in the rates, though that for ages 35–39 started to increase in 1975 and that for ages 40–44 four years later. At ages 45–54 an initial rise has been followed by a fall to about half of the peak rate.

In Scotland the pattern of change is very similar to that for England and Wales. However there is considerable year to year fluctuation in rates because of the small numbers involved.

These changes in registration rates shown in Tables IIa and IIb correspond to the patterns of mortality described earlier.

Since registration data are available for shorter periods than mortality data it is not possible to carry out such a detailed cohort analysis as for the mortality data. However some information is available for cohorts of women born between about 1901 and 1951. The rates are plotted in Figures 4 and 5. The general pattern for both countries is similar to that for the mortality rates; there is a marked increase in rates for successive cohorts of women born in years around 1931, 1941 and 1951.

*Registration rates for carcinoma in situ*

Carcinoma in situ registration rates, based on the same sources as those for invasive cancer, are given in Tables IIIa and IIIb. The data given in these tables are subject to a number of caveats. First, and

**Figure 4** Cohort registration rates for carcinoma of the cervix in England and Wales 1962–1980. Rates are plotted for women registered at specified ages and born in groups of years centred on January 1st 1901, 1911 etc. Most points are based on registration data for 5 years. The first point for all cohorts except 1951 is based on 4 years' data.

**Figure 5** Cohort registration rates for carcinoma of the cervix in Scotland 1961–1980. Rates are plotted for women registered at specified ages and born in groups of years centred on January 1st 1901, 1911 etc. Each point is based on registration data for 5 years.

**Table IIIa** Registrations of carcinoma in situ of the cervix in England and Wales

| | *Rates per million women* | | | | | |
|---|---|---|---|---|---|---|
| | *Age group* | | | | | |
| *Year* | *15–24* | *25–34* | *35–44* | *45–54* | *55–64* | *65+* |
| 1963–65 | 6 | 62 | 121 | 69 | 20 | 11 |
| 1966–68 | 26 | 218 | 363 | 215 | 45 | 14 |
| 1969–71 | 36 | 254 | 270 | 172 | 44 | 12 |
| 1972–74 | 58 | 316 | 271 | 148 | 49 | 14 |
| 1975–77 | 82 | 492 | 355 | 148 | 51 | 17 |
| 1978–80 | 90 | 616 | 420 | 151 | 55 | 15 |

**Table IIIb** Registrations of carcinoma in situ of the cervix in Scotland

| | *Rates per million women* | | | | | |
|---|---|---|---|---|---|---|
| | *Age group* | | | | | |
| *Year* | *15–24* | *25–34* | *35–44* | *45–54* | *55–64* | *65+* |
| 1960–62 | 1 | 79 | 126 | 79 | 24 | 18 |
| 1963–65 | 7 | 133 | 218 | 137 | 40 | 17 |
| 1966–68 | 28 | 316 | 494 | 309 | 60 | 17 |
| 1969–71 | 37 | 307 | 320 | 182 | 62 | 14 |
| 1972–74 | 50 | 374 | 244 | 137 | 40 | 14 |
| 1975–77 | 73 | 481 | 314 | 113 | 41 | 14 |
| 1978–80 | 94 | 568 | 392 | 102 | 39 | 10 |

most obviously, the rates are to a considerable extent a reflection of the numbers of smears taken: in general as the number of smears taken increases the number of 'positive' cases will increase and, since registrations are expressed as rates relative to the total population rather than to the numbers who have been screened, this will appear as an increase in rates. Secondly, it seems fairly certain that not all cases of carcinoma in situ are in fact notified to cancer registries. However it is possible to draw one, admittedly tentative, conclusion from Table IIIa. In England and Wales, between 1972–74 and 1978–80, the number of smears notified to the D.H.S.S. increased by only about 19% – from about 2.31 million to about 2.75 million per year. It is estimated that in 1978–80 more than 60% were examinations for women who had had previous smears (unpublished data provided by D.H.S.S.). In general it is to be expected that the 'positive' rate will decrease with repeated screenings since an initially existing pool of positive cases (sometimes called the 'prevalence cases') will be depleted leaving only false negatives and new (incident) cases to be detected at later screenings. Thus it might be expected that the registration rate for carcinoma in situ would have *decreased* between 1972–74 and 1978–80 since the increase in the number of smears taken was relatively small and in the earlier period a greater propoertion would have been 'first time' smears. Table IIIa shows that in fact at ages 15, 24, 25–34 and 35–44 the rates between 1972–74 and 1978–80 increased substantially particularly among women aged 25–34. This strongly suggests that there has been a true increase in premalignant lesions in recent years. There are alternative explanations: for instance it might be that a greater proportion of high-risk women have come forward for screening in recent years, and that the rates are therefore biased, or that the interval between smears has increased so that there is a greater chance of new lesions arising. We know of no evidence in favour of either of these possibilities and it seems improbable that these factors would affect only younger women and that the rates would have continued to increase. Table IIIb shows similar changes for rates of carcinoma in situ in Scotland.

The high rates shown in Tables IIIa and IIIb for the period 1966–68, particularly for age-groups 35–

44 and 45–54, suggest that large numbers of 'prevalence cases' were picked up in the early stages of the screening programme.

*Relationship between numbers of cervical smears and registrations of carcinoma in situ*

In Tables IVa and IVb we present the numbers of registrations of carcinoma in situ related to estimated numbers of smears taken in each year for various age groups. The numbers of registrations used for these tables are taken from published cancer registration data as explained above. The estimated numbers of smears for England and Wales are based on the summary forms SBH140 submitted to the D.H.S.S. by Area Health Authorities and cervical smear forms sent to the National Health Service Central Register (Roberts, 1982); similar arrangements exist for the Scottish data. Table IVa shows that in England and Wales rates of carcinoma in situ per thousand smears taken have been increasing, the sharpest increase occurring among women aged 25–29 and 30–34. In these two age groups the total number of smears increased by only 11% between 1973 and 1980 whereas the number of cases of carcinoma in situ registered increased by 131%. This again provides evidence that there is a real increase in incidence particularly among younger women. The pattern in Table IVa is similar to that reported by Roberts who showed an increasing rate of 'positive' cases (smears classified cytologically as 'severe dysplasia/carcinoma in situ' or 'carcinoma in situ/? invasive') during the period, this increase again being most marked in age groups 25–29 and 30–34. Of the cytologically positive cases for which a histological diagnosis is available about 50% are classified as carcinoma in situ; this figure changed little during the period covered by Table IVa. (The estimated numbers of cases of carcinoma in situ in the paper by Roberts are substantially greater than the numbers of registrations used in Tables IIIa and IVa of the present paper. This difference may be due to the fact that not all cases are notified to cancer registries. However, the discrepancy may also be due to the methods used in estimating the numbers of cases for Roberts' paper; these estimates are based on statistical returns made to the D.H.S.S., and it is possible that some biopsy findings corresponding to positive smears are counted twice because the smear was repeated; we have no indication of the magnitude of this possible source of error.)

For Scotland we have estimated from unpublished data the annual numbers of smears in each age-group; Table IVb shows that rates of registered cases of carcinoma in situ per thousand smears have increased also in Scotland between 1973 and 1980 for all ages. As in England and Wales the largest increases have occurred in women aged less than 35 years, in which age group the total number of smears increased by 18% during this period whereas the number of cases of carcinoma in situ increased by 112%.

**Table IVa** Registrations of carcinoma in situ of the cervix in England and Wales

| | *Rates per 1000 smears Age group* | | | |
|---|---|---|---|---|
| *Year* | *Less than 25 years* | *25–29* | *30–34* | *35 and over* |
| 1973 | 0.37 | 1.06 | 1.61 | 1.33 |
| 1974 | 0.40 | 1.31 | 1.77 | 1.39 |
| 1975 | 0.42 | 1.50 | 1.82 | 1.48 |
| 1976 | 0.52 | 1.72 | 2.10 | 1.48 |
| 1977 | 0.50 | 1.94 | 2.61 | 1.63 |
| 1978 | 0.53 | 2.05 | 2.64 | 1.43 |
| 1979 | 0.47 | 2.08 | 2.96 | 1.46 |
| 1980 | 0.53 | 2.20 | 3.18 | 1.49 |

**Table IVb** Registrations of carcinoma in situ of the cervix in Scotland

| | *Rates per 1000 smears Age group* | | |
|---|---|---|---|
| *Year* | *Less than 30 years* | *30–34* | *35 and over* |
| 1973 | 0.87 | 1.59 | 1.27 |
| 1974 | 0.74 | 1.80 | 1.45 |
| 1975 | 0.99 | 0.93 | 1.33 |
| 1976 | 1.42 | 2.00 | 1.62 |
| 1977 | 1.16 | 2.73 | 1.35 |
| 1978 | 1.02 | 1.93 | 1.46 |
| 1979 | 1.18 | 2.25 | 1.29 |
| 1980 | 1.73 | 2.44 | 1.63 |

### Discussion

*The effects of the screening programme*

The evidence from mortality rates presented above has been commented on by several previous authors (e.g. Beral, 1974; Yule, 1978; Draper & Cook, 1983). Data showing an increasing rate of positive smears as observed in either family planning clinics or district cytology laboratories have been presented by Bamford *et al.* (1982), Wolfendale *et al.* (1983) and Mackenzie *et al.* (1983).

In our view the only reasonable interpretation of the available data is that there has been among younger women a very substantial increase in the rates of carcinoma in situ. This is evident from the data presented in Tables IIIa, IIIb, IVa and IVb. In view of the known epidemiological features of cervical neoplasia, and the probably increasing exposure to a sexually transmissible agent resulting from changing patterns of sexual behaviour, this increase was predictable. The increasing use of non-barrier methods of contraception (Wright *et al.*, 1978), the possible direct effects of the contraceptive pill (Vessey *et al.*, 1983) and changes in smoking habits may have contributed to the increase, though the effects of these factors are in varying degrees hard to quantify and controversial.

It is not clear to what extent the substantial decreases in the rates for mortality and registrations of invasive cancer of the cervix at ages 35–44 and 45–54 can be attributed to screening, since improvements occurred at different times for different age groups. These reductions might at least partly be explained by differences in exposure to risk for different cohorts.

The reductions could conceivably also be partly explained by the increasing prevalence of hysterectomy performed for reasons other than cervical cancer. The effect of such an increase is effectively to decrease the population at risk, since the appropriate denominator for cervical cancer rates is the number of cervices rather than the number of women. The proportion of women having had a hysterectomy by any given age can be estimated using data from the Hospital Inpatient Enquiry (HIPE), which is based on hospital discharges in England and Wales, as described by Alderson & Donnan (1978). Their calculations have been extended by Parkin *et al.* (submitted for publication) and by the present authors to provide estimates of the percentages of women in each 5-year age-group who had ever had a hysterectomy, for years between 1963 and 1983. There is a pronounced cohort effect in these rates, and at ages between 35 and 69 the rates for 1983 are two or three times as large as those for 1963. The maximum value calculated is for age group 55–59 in 1983, for which it is estimated that the prevalence of hysterectomy is about 14%. However, as Alderson and Donnan pointed out in looking at mortality rates up to 1975, the effect of the adjustment for hysterectomy rates is relatively small. Thus changing hysterectomy rates cannot explain the trends described in the present paper.

If the screening programme has in fact resulted in a reduction in mortality this should be evident in the rates by about the mid-1970s: it would be expected that within any cohort, and using unscreened cohorts as a comparison, the rates for age-groups covered by the screening programme would be lower than would be predicted from their rates at ages pre-dating the screening programme. An examination of Figures 2 and 3, and of the more detailed tables on which they are based, provides some limited support for the suggestion that after making allowance for the low rates in some cohorts there is indeed the predicted effect of screening at times subsequent to the implementation of a widespread screening programme.

Macgregor & Teper (1978) showed that the mortality rates from cervical cancer for women aged 55–64 in those regions of Scotland where more comprehensive screening programmes were introduced declined sharply during the period 1968–76 compared with the rest of Scotland. There appears to be little difference between the various regions in mortality rates for those aged 35–54: all rates have declined. However for those aged 25–34 Macgregor and Teper showed that a small decrease in mortality rates in Scotland became a small increase when the two "well screened" regions were excluded. It should be emphasised that the numbers of cases on which some of their analyses are based are very small.

Parkin *et al.* (submitted for publication) have attempted to estimate the number of cases of invasive cancer of the cervix which would have been registered during one year, 1978, had there been no screening programme in England and Wales. By making various assumptions about the natural history of cervical neoplasia and taking hysterectomy rates into account they have calculated that the level of screening during the fifteen years before that date had prevented up to 25% of the potential cases of invasive disease. Although this is a substantial number it could have been greater if the resources devoted to screening had been more efficiently used since it is well known that the acceptance of screening in England and Wales has been greater among younger women and those of higher social class, and that those at higher risk have not been properly covered by the screening programme.

We conclude that, although the effectiveness of the screening programme in Great Britain has never been evaluated, it appears likely that it has in fact had some impact in reducing mortality and has probably limited the rise among younger women.

### *Future screening policy*

It is difficult to interpret the patterns of rates presented in this paper and to make predictions about future rates. If, as seems likely, the present increase among younger women continues through their lifespan, and if succeeding cohorts show the same and perhaps even a greater increase, the age distributions of both carcinoma in situ and invasive cancer during successive calendar years may show considerable changes. Recent policy guidelines for England and Wales, as described by Draper (1982) and Burslem (1983), explicitly acknowledge that the incidence among younger women may be increasing. However it should be emphasised that the level of screening among younger women should not be unreasonably increased at the expense of neglecting older women. In particular we would emphasise yet again that it is important that any woman over 35 who has not been screened should have at least one smear.

There are still doubts about the most effective strategy for screening and, particularly in view of current trends in incidence, very careful monitoring of the screening effort and the rates of both pre-clinical and clinical disease will be required. A prerequisite for such monitoring is the development of well-designed computerised information systems which will make it possible to record and analyse the necessary data.

We are grateful to the Office of Population Censuses and Surveys and the Information Services Division of the Common Services Agency of the Scottish Health Service for providing us with unpublished data. We thank Mrs Eileen Gunn for typing successive drafts of this paper. The Childhood Cancer Research Group is supported by the Department of Health and Social Security and the Scottish Home and Health Department.

### References

ALDERSON, M. & DONNAN, S. (1978). Hysterectomy rates and their influence upon mortality from carcinoma of the cervix. *J. Epidemiol. Community Health*, **32**, 175.

ARMSTRONG, B. & HOLMAN, D. (1981). Increasing mortality from cancer of the cervix in young Australian women. *Med. J. Aust.*, **1**, 460.

BAMFORD, P.N., BARBER, M. & BEILBY, J.O.W. (1982). Changing pattern of cervical intraepithelial neoplasia seen in a family planning clinic. (Letter). *Lancet*, **i**, 747.

BARRETT, J.C. (1973). Age, time and cohort factors in mortality from cancer of the cervix. *J. Hygiene* (Cambridge). **71**, 253.

BERAL, V. (1974). Cancer of the cervix: A sexually transmitted infection? *Lancet*, **i**, 1037.

BOURNE, R.G. & GROVE, W.D. (1983). Invasive carcinoma of the cervix in Queensland: Change in incidence and mortality, 1959–1980. *Med. J. Aust.*, **1**, 156.

BURSLEM, R.W. (1983). Cervical cytological screening for users of oral contraceptives. (Letter) *Lancet*, **ii**, 968.

CASE, R.A.M. (1956). Cohort analysis of mortality rates as an historical or narrative technique. *Br. J. Prev. Soc. Med.*, **10**, 159.

DRAPER, G.J. (1982). Screening for cervical cancer: revised policy. The recommendations of the DHSS Committee on Gynaecological Cytology. *Health Trends*, **14**, 37.

DRAPER, G.J. & COOK, G.A. (1983). Changing patterns of cervical cancer rates. *Br. Med. J.*, **287**, 510.

GREEN, G.H. (1979). Rising cervical cancer mortality in young New Zealand women. *NZ. Med. J.*, **89**, 89.

HILL, G.B. & ADELSTEIN, A.M. (1967). Cohort mortality from carcinoma of the cervix. *Lancet*, **ii**, 605.

MACGREGOR, J.E. & TEPER, S. (1978). Mortality from carcinoma of cervix uteri in Britain. *Lancet*, **ii**, 774.

MACKENZIE, E.F.D. & SIMPSON, D. (1983). Changing patterns of cervical cancer rates (Letter). *Br. Med. J.*, **287**, 912.

OFFICE OF POPULATION CENSUSES AND SURVEYS. (OPCS) (1982). *Cancer Statistics: Survival 1971–75 Registrations, England and Wales.* (Series MB1, No. 9). London, HMSO.

OSMOND, C., GARDNER, M.J. & ACHESON, E.D. (1982). Analysis of trends in cancer mortality in England and Wales during 1951–80 separating changes associated with period of birth and period of death. *Br. Med. J.*, **284**, 1005.

OSMOND, C., GARDNER, M.J. & ACHESON, E.D. (1983). *Trends in Cancer Mortality: Analyses by Period of Birth and Death, 1951–1980, England and Wales.* (Series DH1 no 11.) London, HMSO.

REPORT OF A TASK FORCE reconvened by the Health Services Directorate, Health Services and Promotion Branch (1982). *Cervical Cancer Screening Programmes 1982.* Ottawa: Health and Welfare, Canada.

ROBERTS, A. (1982). Cervical cytology in England and Wales, 1965–80. *Health Trends*, **14**, 41.

VESSEY, M.P., LAWLESS, M., MCPHERSON, K. & YEATES, D. (1983). Neoplasia of the cervix uteri and contraception: a possible adverse effect of the pill. *Lancet*, **ii**, 930.

WOLFENDALE, M.R., KING, S. & USHERWOOD, M. McD. (1983). Abnormal cervical smears – are we in for an epidemic? *Br. Med. J.*, **287**, 526.

WRIGHT, N.H., VESSEY, M.P., KENWARD, B. MCPHERSON, K. & DOLL, R. (1978). Neoplasia and dysplasia of the cervix uteri and contraception: a possible protective effect of the diaphragm. *Br. J. Cancer*, **38**, 273.

YULE, R. (1978). Mortality from carcinoma of the cervix. *Lancet*, **i**, 1031.